# Tubulin TUBB4B Is Involved in Spermatogonia Proliferation and Cell Cycle Processes

**DOI:** 10.3390/genes13061082

**Published:** 2022-06-17

**Authors:** Meiying Feng, Kai Wang, Shuying Fu, Hengxi Wei, Xiaokun Mu, Li Li, Shouquan Zhang

**Affiliations:** 1 College of Life Science, Zhaoqing University, Zhaoqing 526061, China; jony.ya@163.com (M.F.); bifushuying@mail.scut.edu.cn (S.F.); mythbelife@163.com (X.M.); 2 National Engineering Research Center for Breeding Swine Industry, Guangdong Provincial Key Lab of Agro-Animal Genomics and Molecular Breeding, College of Animal Science, South China Agricultural University, Guangzhou 510642, China; weihengxi@163.com (H.W.); lili007@scau.edu.cn (L.L.); 3 College of Animal Science and Technology, Nanjing Agricultural University, Nanjing 210095, China; wk@stu.scau.edu.cn

**Keywords:** *Tubb4b*, mouse, spermatogonia cell, CRISPR/Cas9, cell cycle

## Abstract

*Tubb4b* (tubulin β-4b chain) is essential for cell growth and development as a microtubule network protein. Previous studies have shown that TUBB4B affects mouse pronucleus migration, but the gene function has yet to be elucidated. To study TUBB4B-related functions in mouse reproductive development, we designed a single sgRNA in chromosome 2 and generated a knockout spermatogonia cell line of the β-tubulin isoform *Tubb4b* by the CRISPR/Cas9 system. *Tubb4b*-KO spermatogonia recognized abnormal lysosomal membranes and cell morphology defects. Compared to control mouse spermatogonia, the proliferation rate was significantly slower and cycling stagnated in the G1/0 population. Although spermatogonia lacking TUBB4B have abnormal divisions, they are not lethal. We detected the mRNA levels of the cell-regulating cyclins *CyclinsD1*, *CyclinsE*, *Cdk2*, *Cdk4*, *P21*, *Skp2* and the cell growth factors *C/EBP α*, *C/EBP β*, and *G-CSF* in the spermatogonia of *Tubb4b*-KO and found that the expressions of *CyclinsD1*, *Skp2* and cell growth factors were significantly reduced. Further analysis revealed that 675 genes were expressed differently after *Tubb4b* deletion and were enriched in negative regulation of cell population proliferation (GO:0008285), negative regulation of cell cycle G2/M phase transition (GO:1902750), and positive regulation of cell death (GO: 0010942). We also found that there is a common gene Cdkn1a (P21) in these three GO pathways related to cell proliferation and cell cycle, and both quantitative analysis and transcriptome sequencing results showed that the expression of this gene was up-regulated in *Tubb4b* knockout cells. This implies that *Tubb4b* may be involved in the division of spermatogonia with multiple cell cycle regulatory proteins. Overall, these data indicate that *Tubb4b* has a specific role in regulating spermatogonia proliferation and cell cycle.

## 1. Introduction

The timely and orderly expression of testis-specific genes is a critical step in the spermatogenesis and maturation process. The ordered transport and integration of the skeletal structure of spermatozoa and related proteins drives the coordinated synthesis and assembly of various organelles in sperm cells to ensure normal function of sperm [1]. In the process of spermatogenesis, microtubules are expressed at multiple developmental stages. During the development of sperm cells, microtubules are first enriched around the sperm nucleus, and then the microtubule expression of sperm continues to increase, promoting the formation of the manchette. The short-lived actin-containing structure is essential for shaping the nucleus and the head of sperm and the construction of the tail. With the progress of spermatogenesis, α-tubulin and β-tubulin extend from the centrosome to a sperm tail and are arranged in a unique structure with other components of the flagellar axon. This structure directly controls the normal development of sperm and sports form [2]. Studies have shown that microtubule defects may lead to abnormal male reproduction, especially abnormal expression of β-tubulin, which has been reported in many studies related to sperm development. The molecular weight of the β-tubulin protein is 55KD. It is an acidic polypeptide composed of 447 amino acids. It exists on the lower edge, neck, and tail of sperm and plays an essential role in sperm movement and fertilization. Radiation has been reported to reduce sperm motility and β-tubulin protein expression in adolescent mice, impairing sperm flagellar motility [3,4]. Furthermore, studies have pointed out that the abnormal expression of β-tubulin protein in human and wild boar sperm heads affects the transition of the initial stage of fertilization [5]. Under pathological conditions, the distribution of acrosome protein and β-tubulin in the sperm head changes significantly, and their mRNA and protein expression are also reduced, which seriously affects fertilization functions [6]. After observing that the abnormal expression of β-tubulin affects sperm development, some studies have performed some essential characterization of the family genes that make it up.

The TUBB4B protein belongs to the β-tubulin gene family and is the main component of microtubules that influence the processes of pronucleus migration in mice [7,8]. β-tubulin has eight isotype structures: TUBB, TUBB2A, TUBB2B, TUBB3, TUBB4, TUBB2C, Tub6, TBB1a microtubules. The proteins form heterodimers that make up the precursor filaments of microtubules [2]. These hollow tubes are a key component of the cytoskeleton and are involve in the dynamic structure of many cellular processes. A complete microtubule network is essential for normal spermatogenesis. Analyse of its molecular function is possible through biosynthesis enriched with the structural components of double-stranded RNA binding GTPase, molecular activity, GTP binding and the cytoskeleton. The biological processes involved are the organization of the microtubule cytoskeleton and the mitotic cell cycle. Changes in post-translational modifications within tubulin subunits, such as phosphorylation, may regulate the interaction between microtubules and microtubule-associated protein (MAP), thereby affecting the dynamic instability of microtubules. In interphase cells, microtubules are located near the nucleus, in the centrosome (negative end), and radiate out to the cell periphery (positive end), maintaining cell shape and cell-cell interaction, cell-matrix adhesion, protein transport, and cell movement. Furthermore, microtubules form a mitotic spindle that enables correct chromosome separation during cell division. Therefore, they can be one of the main targets of cancer treatments that potentially prevent cell proliferation [9]. The regulatory effects of changes in TUBB3 and TUBB4B levels on cancer progression have been studied [10]. By analysing the regulation of TUBB3 and TUBB4B levels in the development of non-small cell lung cancer and prostate cancer, we understand the characteristic mechanism of cancer cells’ reduced sensitivity to vinca alkaloids and taxanes, and other tubulin-binding agents (TBA) [11,12]. In addition, TUBB3, TUBB4B, and TUBB6 were down-regulated in taxane-resistant breast cancer cells [13]. Pilatz et al. found that the expression of the TUBB4B protein in the sperm of patients with acute orchitis decreased [14]. It is reported that the TUBB4B mutation has a significant inhibitory effect on normal microtubules growth, and the expression level of TUBB4B is crucial for microtubule-vimentin interaction and maintaining the polarity of migrating cells. Cells with low TUBB4B concentrations have a faster aggregation rate of microtubules. These studies also point out that the abnormal expression of the TUBB4B protein seems to be related to cell division and proliferation [15].

Although research and functional analysis of *Tubb4b* suggest that it appears to be related to sperm development and cell division, the research only deals with changes in TUBB4B protein expression during reproductive development, but the function of *Tubb4b* on germ cells is unclear. The sequence similarity of mouse and human TUBB4B protein was 100% [16]. We knocked out *Tubb4b* in mouse spermatogonia by CRISPR-cas9. The effects of *Tubb4b* on spermatogonia proliferation, the cell cycle and the expression of related cycle regulatory proteins were demonstrated, and the changes in cell structure were observed with an electron microscope. We found that deletion of *Tubb4b* caused destruction of cell structure and affected spermatogonia proliferation and cycling through the expression of a series of cyclins. This study aimed to determine the effect of *Tubb4b* on the prophase of sperm development at the cellular level and to explore the mechanism.

## 2. Materials and Methods

### 2.1. Cell Culture

The mouse spermatogonia from C57 mice were obtained from Prof. Wenxian Zeng (School of Animal Science and Technology of Northwest A&F University, Xianyang, China), and cultured with 89% DMEM (C11995500BT, Invitrogen, Waltham, MA, USA), 10% FBS (FSP500, ExCell, Shanghai, China), and 1% L-glutamine (25030, Invitrogen, Waltham, MA, USA). A 0.25% trypsin-EDTA solution (25200072, Invitrogen, Waltham, MA, USA) of 2 mL was added to the cells when their density reached 80%~90% and digested for about 3 min before the complete medium was added to terminate the experiment. Afterward, the cell suspension was centrifuged at 1100 rpm for 3 min, then cells were cultivated in a petri dish containing 2 mL complete medium with 5% CO_2_.

### 2.2. Amplification of Target Sequence Fragments and Liposomes Transfection

For defining the specificity target in *Tubb4b*, the target sequence was predicted in the second exon of the *Tubb4b* on the mouse genome according to the method provided by Zhang Feng’s laboratory [17] and the specificity of target was assessed by mapping it to the mouse genome chromosome via the Ensembl BLAST Tool (http://asia.ensembl.org/Multi/Tools/Blast?db=core/; accessed on 11 August 2020). Following that, the specific primers designed by Primer 5.0 software were employed to amplify the mU6 fragment and the SgRNA sequence fragment, respectively, of the target sequence using the plasmid vector as the template. To obtain the mU6-Tubb4b-SgRNA fragment, the mU6 promoter sequence and the Tubb4b-SgRNA target sequence were fused by overlapping PCR amplification utilizing the sense primer of mU6 and the antisense primer of SgRNA (The primer sequences are shown in Supplementary Table S1). Finally, 1 μg mU6-Tubb4b-SgRNA fragment was transfected into Cas9-expressing mouse spermatogonia, which stably expressed *Cas9*, with Lipofectamine3000 transfection reagent (L3000-015, Thermo Fisher, Waltham, MA, USA). After 24 h, doxycycline (D9891, Sigma, Shanghai, China) was given to induce CAS9 protein expression. After 48 h, the cell genome was extracted to do a template, and the fragments near the target sequence of *Tubb4b* were amplified with genome primers and the 2 × Taq Master Mix (P111, Vazyme, Nanjing, China) by RT-PCR. The PCR conditions were a single cycle of 94 °C for 5 min, then followed by 35 cycles of 94 °C for 30 s, 60 °C for 30 s, and extension at 72 °C for 1 min, and last, a single cycle of 72 °C for 7 min. The PCR products were reclaimed allowing for 3% agarose gel electrophoresis and examined by base sequencing to analyse the cleavage activity via the SeqMan software.

### 2.3. The Lentivirus Packaging and Infection of pLVX- mU6-Tubb4b-SgRNA-EGFP

To construct the pLVX-mU6-Tubb4b-SgRNA-EGFP vector, the cleavage-active target sequence and the empty vector (pLVX-EGFP) were digested by *Nhe* I (1241A, Takara, Guangzhou, China) and *Xho* I (1094A, Takara, Guangzhou, China), and connected together using T4 DNA Ligase (2011A, Takara, Guangzhou, China). Then *Nhe* I and *Xho* I digestion was used to confirm the vector’s assembly. After that, lentivirus packaging was performed as antecedently described [18] to obtain Tubb4b-SgRNA virus, and Polybrene (H9268, Sigma, Shanghai, China) at a final concentration of 6 μg/mL was added to the culture medium to assist infect the mouse spermatogonia.

After 12 h, the lentivirus was removed and mouse spermatogonia were selected with 10 µg·mL-1 blasticidin (B9300, Solarbio, Beijing, China) for 5 days to 7 days to gain mouse spermatogonia stably expressing Tubb4b-SgRNA. The fluorescence of mouse spermatogonia following infection may then be seen with a fluorescence microscope (TH4-200, Olympus).

### 2.4. Flow Cytometry Analysis

To detect the proportion of fluorescent cells, the cells were digested into single cells with 0.25% trypsin-EDTA solution, which was processed by the above method and washed three times with DPBS (C14190500BT, Invitrogen, Waltham, MA, USA) by centrifuging at 1100 rpm for 3 min. Then flow cytometry (CytoFLEX S, Beckman Coulter, Suzhou, China) was observed to detect the number of mouse spermatogonia to calculate the proportion of fluorescent cells between Tubb4b-SgRNA virus infection (*Tubb4b*-KO) and the control of mouse spermatogonia. Finally, using the original mouse spermatogonia as controls, cell apoptosis and cell cycle analysis were performed by flow cytometry as previously described [18].

### 2.5. PCR Amplification and pMD18-T Vector Connection

The *Cas9* sequence and the *Tubb4b* sequence near the target gene were amplified according to the above method with *Cas9* and the *Tubb4b* genome primers (The primer sequences are shown in Supplementary Table S1). Subsequently, the amplified *Tubb4b* fragment was reclaimed allowing for 3% agarose gel electrophoresis and examined by base sequencing. The results of the sequencing were compared with the sequence published by NCBI, and the effect of *Tubb4b* knockout in mouse spermatogonia was analysed using Lasergene software according to the base peak map. At the same time, the amplified *Tubb4b* fragment was connected to the pMD18-T Vector (6011, Takara, Guangzhou, China), then the positive colonies, which were connected to the DNA fragment through gene cloning and PCR amplification (universal primer M13), were obtained and were sent to the company for base sequencing analysis. Next, using the *Tubb4b* mouse genome as a standard, the sequencing results were analysed by Lasergene software to calculate the knockout efficiency of the *Tubb4b* gene.

### 2.6. Western Blot Detection

Following the previous method [18], a whole-cell lysis assay (KGP250, KeyGEN BioTECH, Nanjing, China) and the BCA Protein Quantitation Assay (KGPBCA, KeyGEN BioTECH, Nanjing, China) were used to extract the total protein and detected the protein concentration from the control mouse spermatogonia or from *Tubb4b*-KO mouse spermatogonia. Moreover, all of the protein concentration samples were adjusted to 1 μg·μL^−1^ by 2× Laemmli Sample Buffer (1610737, Bio-Rad, Hercules, CA, USA), after that the processes of protein electrophoresis (4% concentrated gel constant pressure 70 V for 40 min and 10% separation gel constant pressure 90 V for 2 h), membrane transfer (using polyvinylidene difluoride (PVDF) membranes (Hybond-P, Helsinki, Finland) and 80 V pressure for 1 h), blocking of skim milk (232100, BD, USA), antibody incubation, Pro-light HRP chemiluminescence (PA112, TIANGEN, Beijing, China) and automated chemiluminescence analysis (Tanon5200, Tianneng) were used to analyse the expression of the CAS9, TUBB4B proteins. Among them, the antibodies and dilution ratios used for analysis were as follows: a mouse monoclonal anti-ACTIN antibody (HC201, TransGen Biotech, 1:1000), a mouse monoclonal anti-FLAG antibody (1:1000), a mouse monoclonal anti-GAPDH antibody (E021010, Canlife, 1:1000), a rabbit monoclonal anti-TUBB4B antibody (ab179512, Abcam, 1:1000), HRP Affini Pure Goat Anti-Mouse IgG(H + L) (E030110, Earth Ox, 1:10,000), HRP Affini Pure Goat Anti-Rabbit IgG(H + L) (E030120, EarthOx, 1:10,000).

### 2.7. The Cells Growth Curve

Control and *Tubb4b*-KO mouse spermatogonia were lysed into a single cell suspension by 0.25% Trypsin-EDTA and counted by a cell count plate (177-112C, Watson, Japan). After counting, 5000 cells were seeded in a 96-well plate (4 replicate wells per group) and cultured at 37 °C in a 5% CO_2_ atmosphere. The next day, the cells were mixed with 20 μL MTS reagent (G3582, Promega, Madison, WI, USA) per well, incubated for 1 h in the dark and the absorbance measured at a wavelength of 490 nm in a microplate reader (Synergy2, BioTek) to evaluate the cell viability, which is also recorded as the day 1. For the next 4 consecutive days, the cell absorbance values were measured periodically at the same time and recorded on the 2nd day, 3rd day, 4th day, and 5th day, respectively. Finally, Microsoft Excel software was used to perform a *t*-test analysis of the absorbance value and plot the cell growth curve.

### 2.8. Real-Time Fluorescence Quantitative PCR Analysis

Lysing control and *Tubb4b*-KO mouse spermatogonia into a single cell suspension again, and the total RNA extracted and cDNA synthesis of control or *Tubb4b*-KO mouse spermatogonia were performed as previously reported [18]. Then, the Ct values of the target genes were measured with AceQ Universal SYBR qPCR Master Mix (Q511-02, Vazyme, Nanjing, China) on the Bio-Rad CFX96 PCR system (CFX96, USA), and the relative expression level was used to compare gene expression between the control and *Tubb4b*-KO mouse spermatogonia. Among them, *Gapdh* was used as the internal reference gene to calculate the ΔCt value (ΔCt = target gene Ct-internal reference gene Ct), the ΔΔCt method (ΔΔCt = ΔCt (test group) − ΔCt (control group), relative expression = 2^−ΔΔCt^) was used to calculate the relative expression of the target gene. Finally, the quantitative results were analysed by Microsoft Excel software with *t*-test analysis (The primer sequences are shown in Supplementary Table S1).

### 2.9. Immunofluorescence Detection

First, the control and *Tubb4b*-KO spermatozoa cells were fixed and ruptured membranes with DPBS, including 4% paraformaldehyde (P0099, Beyotime, Shanghai, China) 0.1% Triton X-100 (X100, Sigma, Shanghai, China) for 30 min. After washing 3 times with general DPBS, the cells were blocked in 1% BSA (A8806, Sigma, Shanghai, China) for 30 min. Then, cells were incubated with a mouse monoclonal anti-β-Tubulin antibody (T8328, Sigma, 1:1000) at 4 °C overnight. The next day, using general DPBS to wash 3 times again, the secondary antibody, Alexa Fluor 568-labeled goat anti- mouse IgG (A11031, Invitrogen, 1:100) was incubated at 37 °C in the dark for 1 h. Finally, continuing the general DPBS washing, cells were added 10 μg/mL Hoechst 33342 (62249, Thermo Scientific, Waltham, MA, USA) to stain the nuclei and observed the fluorescence under the fluorescence microscope.

### 2.10. The Data Analysis of Transcriptome Sequencing

A total of 1 μg RNA per sample was used for the RNA sample, and then the sequencing libraries were generated by NEBNext UltraTM RNA Library Prep Kit for Illumina (E7530S, NEB, Beijing, China). After the samples were purified with the AMPure XP system (A63881, Beckman Coulter, Brea, CA, USA), PCR amplification was performed, and PCR products were purified. Finally, the libraries were sequenced on the Illumina HiSeq 2500 platform (Illumina, Inc., San Diego, CA, USA) using fragments 240 bp in length.

For further research, raw data (raw reads) of fastq format were processed to clean data (clean reads) by removing reads containing adapter, reads containing ploy-N and low quality read. Then the clean reads were mapped to the reference genome sequence by the Tophat2 tool, and the DESeq R package (4.0.2) was used to analyse the differential expression of two conditions/groups with a FDR < 0.05 after that the FDR were adjusted the false discovery rate using Benjamini and Hochberg’s.

### 2.11. Analysis of the Differentially Expressed Genes

As previously described, the DEGs, which have the same or similar expression patterns, were directly reflected in the cluster analysis conducted using the hierarchical complete linkage clustering method in R software (R version 3.4.3) [7]. The Gene Ontology (GO) enrichment analysis of the DEGs was implemented by Metascape (https://metascape.org/, accessed on 8 April 2022), which can map the DEGs into mice based on both EggNOG and Homologene databases [19]. Finally, the sequences of the DEGs were blast (blastx) searched to the mouse genome using the STRING database (http://stringdb.org/, accessed on 8 April 2022) to get the predicted PPI (protein-protein interaction) of these DEGs, and Cytoscape V3.6.1 software was used to construct the network of protein interaction based on the export data from the STRING database [20].

### 2.12. Statistical Analysis

All experiments were repeated at least three times. The statistical significance of the differences between groups was determined using the *t*-test on Microsoft Excel software. The results are expressed as the means ± standard deviation (SD).

## 3. Results

### 3.1. Tubb4b-SgRNA Target Gene Screening and Lentiviral Shuttle Vector Construction

**Figure 1:**
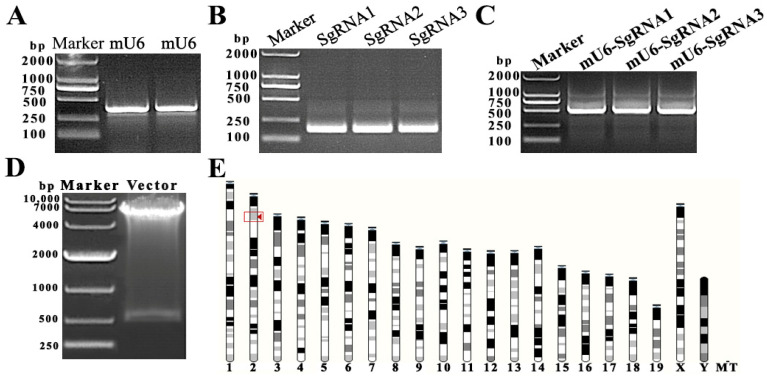
Construction of lentiviral shuttle vector pLV-mU6-Tubb4b-SgRNA-EGFP. (**A**): amplification of mU6 fragment (375 bp); (**B**): *Tubb4b* target sequence and the amplification of SgRNA fragment (163 bp); (**C**): mU6-Tubb4b-SgRNA sequence (518 bp) amplification; (**D**): plvx-mU6-Tubb4b-SgRNA-blast-EGFP lentiviral shuttle vector double enzyme digestion (*Nhe* I and *Xho* I) verification; (**E**): Tubb4b-sgRNA target sequence in the chromosomal position of the mouse genome.

The knockout activity of the SgRNA target sequence is critical to the results of the experiment. We amplified the mU6 promoter sequence (Figure 1A) and the three predicted Tubb4b-SgRNA sequences (Figure 1B) expected to have knockout activity in the second exon of the *Tubb4b* genome and linked them to mU6 by overlapping PCR to form Tubb4b-SgRNA fragments (Figure 1C). Thereafter, the three fragments were transfected into mouse spermatogonia by the liposome transgene method, and the target sequence of Tubb4b-SgRNA CCGGTAAGGCTCCTCTACCC with cleavage activity was screened by RT-PCR technology and base sequencing (results not shown). The active mU6-Tubb4b-SgRNA targeting sequence with CCGGTAAGGCTCCTCTACCC was ligated to the pLVX-EGFP-SgRNA lentiviral shuttle vector to obtain the pLVX-EGFP-Tubb4b-SgRNA vector. After *Nhe* I and *Xho* I digestion to verify the vector construction results (Figure 1D), we successfully obtained the lentiviral shuttle vector pLVX-EGFP-Tubb4b-SgRNA. In addition, it was determined that the target sequence had a unique position in the mouse genome and was located on chromosome 2 (Figure 1E), after comparing the Tubb4b-SgRNA activity sequence to the mouse genome.

### 3.2. Screening of Mouse Spermatogonial Cell Lines of Tubb4b-KO

**Figure 2:**
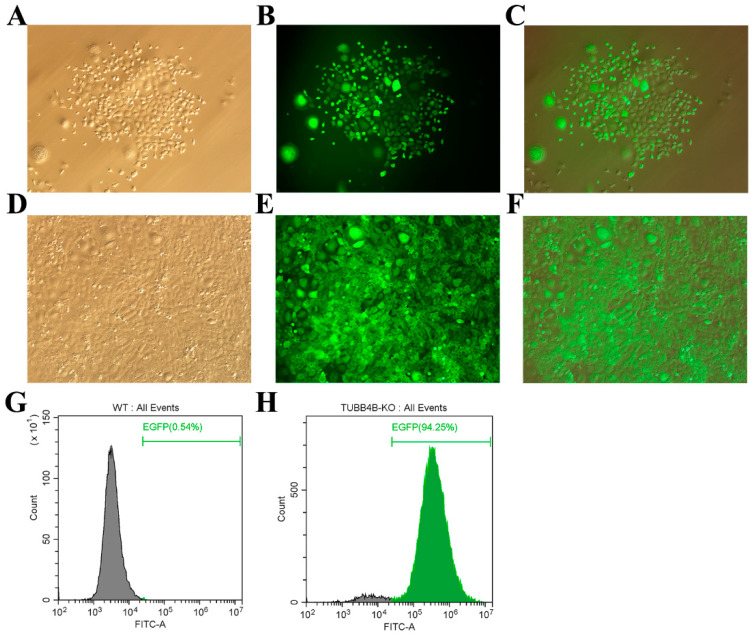
TUBB4B-KO mouse spermatogonia screening 100×. (**A**,**B**): White light and fluorescence images of mouse spermatogonia selected after virus infection; (**C**): Merge results of (**A**) and (**B**). (**D**,**E**): White light and fluorescence of mouse spermatogonia of TUBB4B-KO; (**F**): Merge result of (**D**,**E**). (**G**): Flow cytometry to detect the expression of fluorescent protein in control mouse spermatogonia; (**H**): Flow cytometry to detect the expression of fluorescent protein in *Tubb4b*-KO mouse spermatogonia. Note: Green numbers indicate the proportion of cells expressing EGFP protein in control or *Tubb4b*-KO mouse spermatogonia.

Lentivirus packaging and infection technology can achieve stable integration of foreign genes into animal cells. We co-transfect 293 FT cells with lentiviral packaging plasmids (pMD2.G and psPAX2) and lentiviral shuttle plasmids (pLVX-EGFP-Tubb4b-SgRNA) in a 1:3:3 ratio to obtain Tubb4b-SgRNA virus solution. After 24 h, vector transfection efficiency was found to be greater than 90%. The virus fluid was collected and concentrated after 8 h and 72 h, and the virus titre of the Tubb4b-SgRNA virus liquid was determined by the fluorescence method to reach 10^10^ TU/mL (results not shown). Subsequently, the concentrated Tubb4b-SgRNA virus was infected with mouse spermatogonia, and Blast was added to the screen to obtain positive cell clusters (Figure 2A–C). After expanded culture, mouse spermatogonia cells stably expressed with mU6-Tubb4b-SgRNA can be obtained (Figure 2D–F). Finally, the number of positive cells detected by flow cytometry showed that control mouse spermatogonia without Tubb4b-SgRNA transfer were used as controls, and the positive rate of mouse spermatogonia cells with Tubb4b-SgRNA transfer reached 94.25% (such as Figure 2G,H).

### 3.3. Tubb4b Gene Knockout Test

**Figure 3:**
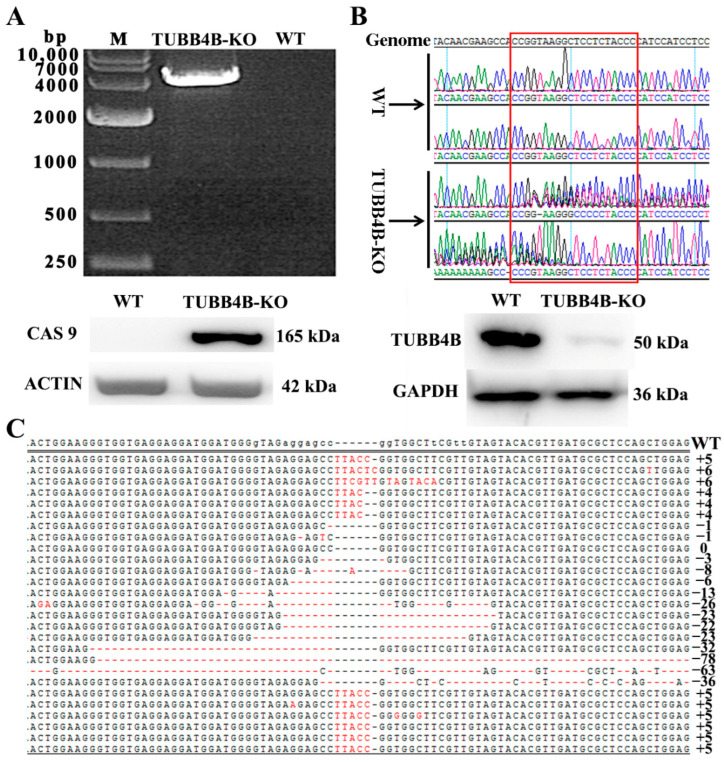
Detection of *Tubb4b* knockout in mouse spermatogonia. (**A**): RT-PCR detection of Cas9 gene expression (**Top**), and western blot detection of CAS9 protein expression in control and TUBB4B-KO mouse spermatogonia (**Bottom**); (**B**): The results of genome sequencing analysis a (Top), and western blot detection of TUBB4B protein expression in control and TUBB4B-KO mouse spermatogonia (Bottom); (**C**): TUBB4B-KO mouse spermatogonia *Tubb4b*-KO efficiency analysis (92.3%). Note: WT represents the control group of control mouse spermatogonia, and TUBB4B-KO represents the test group of TUBB4B-KO mouse spermatogonia, same below. M means marker. The genome represents the mouse genome sequence of TUBB4B, and the red rectangle indicates the SgRNA target of *Tubb4b* in (**B**). The right-hand numbers in (**C**) represent the bases of inserted or deleted, and the ‘+’ is an increase while the ‘−’ is a decrease.

The CAS 9 protein can cut the genome in mouse spermatogonia. After analysing the RNA and protein of control and experimental mouse spermatogonia using RT-PCR and Western-blot technology, we found that it expressed the RNA and protein of Cas9 in the test group mouse spermatogonia of Tubb4b-SgRNA transferred, while the control mouse spermatogonia do not find the expression of them (Figure 3A). To detect the *Tubb4b* knockout effect of mouse spermatogonia, we amplified the 200 bp before and after the target gene for base sequencing and analysed the peak. The results showed that the test group found a double peak, while the control group had only one peak (Figure 3B, top), indicating at the genetic level that we achieved *Tubb4b* gene knockout in mouse spermatogonia (*Tubb4b*-KO). Western blot results also confirmed that we successfully knocked out TUBB4B expression at the protein level (Figure 3B, bottom). To further analyse the knockout efficiency, we further amplified the 200 bp before and after the target gene and ligated into the pMD18-T vector. After combining molecular cloning and analysis of sequencing data, it was found that the knockout efficiency of *Tubb4b* reaches more than 90% (Figure 3C). These data results confirm that we have successfully obtained *Tubb4b*-KO mouse spermatogonia.

### 3.4. Tubb4b-KO Affects the Cell Proliferation of Mouse Spermatogonia

**Figure 4:**
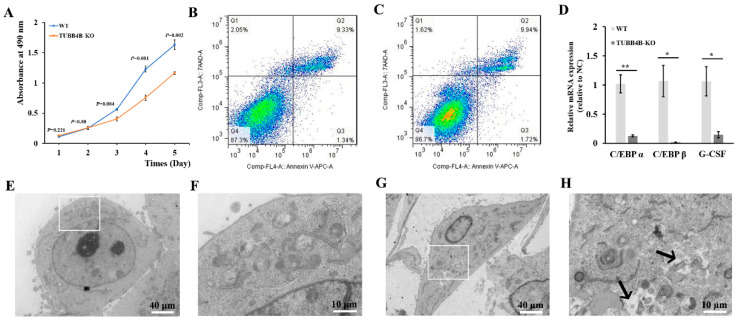
Loss of *Tubb4b* affects the proliferation of mouse spermatogonia. (**A**): Cell growth curve of control and TUBB4B-KO mouse spermatogonia. The *p*-values were obtained by *t*-test comparing with the TUBB4B-KO and control spermatogonia; (**B**): Apoptosis detection of control mouse spermatogonia; (**C**): Detection of apoptosis in spermatogonia of TUBB4B-KO mouse spermatogonia; (**D**): Gene expression of control and TUBB4B-KO mouse spermatogonia on cell proliferation. ‘*’ means 0.01 ≤ *p*-value < 0.05, while ‘**’ means 0.001 ≤ *p*-value < 0.01; (**E**): WT cell ultrastructural. The magnification was 1200×, while the scale bar was 40 μm. And the white rectangles indicate the region of (**F**); (**F**): Enlarged view of (**E**). The magnification was 5000×, while the scale bar was 10 μm; (**G**): *Tubb4b*-KO cell ultrastructural. The magnification and scale bar were the same as in (**E**), and the white rectangles indicate the region of (**H**); (**H**): Enlarged view of (**G**). The magnification and scale bar were the same as in Figure 4F.

At the beginning of the study of *Tubb4b*-KO mouse spermatogonia, it was found that the cells grew slowly after *Tubb4b* knockout. After drawing the growth curve, it was found that the growth rate of mouse spermatogonia from *Tubb4b*-KO was slower than that of control cells (Figure 4A). Thereafter, when the apoptosis was detected by flow cytometry, there was no apparent apoptosis in mouse spermatogonia after *Tubb4b*-KO compared to control mouse spermatogonia (Figure 4B,C); however, qRT-PCR results showed that *Tubb4b*-KO significantly reduced expression of genes *C/EBP α* (*p* < 0.01), *C/EBP β* (*p* < 0.05) and *G-CSF* (*p* < 0.05) in mouse spermatogonia (Figure 4D). *Tubb4b*-KO spermatogonia demonstrated abnormal lysosomal membrane (Figure 4E–H).

**Figure 5:**
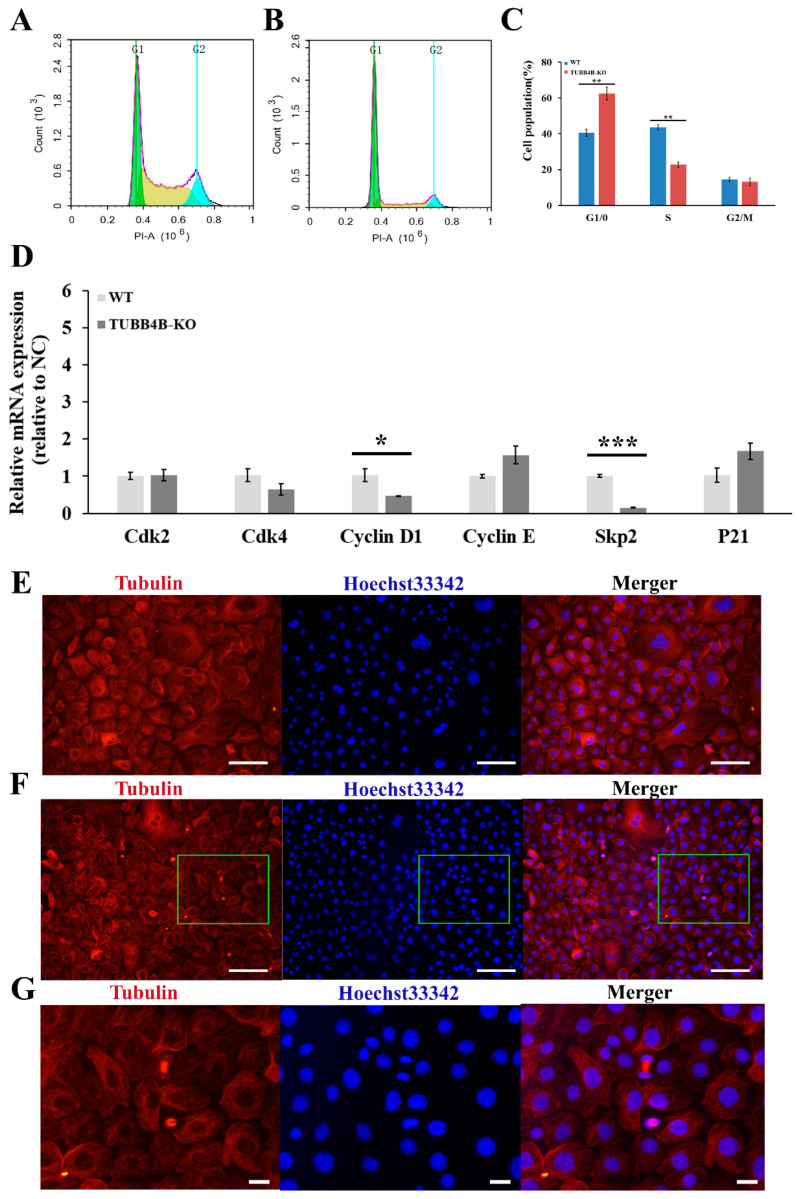
The effect of TUBB4B-KO on mouse spermatogonia cell cycle. (**A**): Cell cycle detection of control mouse spermatogonia by flow cytometry; (**B**): Cell cycle detection of TUBB4B-KO mouse spermatogonia by flow cytometry; (**C**): Cell cycle analysis of control and TUBB4B-KO mouse spermatogonia; (**D**): The cell cycle gene expression between control and TUBB4B-KO mouse spermatogonia; (**E**): immunofluorescence to detect the expression of β-Tubulin protein in control mouse spermatogonia. The magnification was 200×, while the scale bar was 100 μm; (**F**): immunofluorescence to detect the expression of β-Tubulin protein in TUBB4B-KO mouse spermatogonia. The magnification and scale bar were the same as in €, and the green rectangles indicate the region of (**G**); (**G**): The zoomed-in (**F**) part. The scale bar was 20 μm. Furthermore, the G1 means the G0/G1 phase of the cell cycle, the G2 means the G2/M phase of the cell cycle. ‘*’ means 0.01 ≤ *p*-value < 0.05, ‘**’ means 0.001 ≤ *p*-value < 0.01, and ‘***’ means *p*-value < 0.001.

Upon further detection of the cell cycle by flow cytometry, it was shown that the cell cycle changed significantly after *Tubb4b*-KO (Figure 5A,B), which significantly increased the proportion of mouse spermatogonia in the G0/1 phase (*p* < 0.01) and reduced the proportion of mouse spermatogonia in S-phase (*p* < 0.01) (Figure 5C). The expressions of *CyclinsD1* and *Skp2* were significantly decreased in the cells of which *Tubb4b* was knocked out (Figure 5D). The immunofluorescence results showed that the cell number of *Tubb4b*-KO spermatogonia was greater than that of the control cells in the dividing phase (Figure 5E,F). Though most control cells entered the intercellular phase, the *Tubb4b*-KO mouse spermatogonia were still in the dividing phase (Figure 5G). This implies that knocking out *Tubb4b* can inhibit the proliferation of mouse spermatogonia.

### 3.5. Functional Analysis of Differential Gene Expression in Tubb4b-KO Spermatogonia

**Figure 6:**
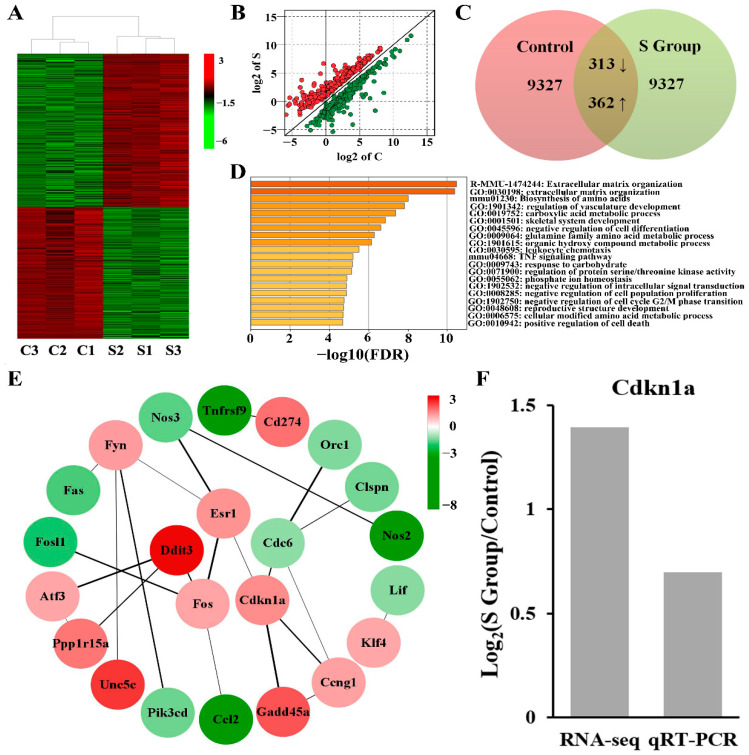
The *Tubb4b* deletion affects gene expression on mouse spermatogonia. (**A**): The clustered heatmaps of DEGs between mouse spermatogonia in the control and experimental groups. The letter C means the control group, while the letter S means the experimental group, same below. And red indicates high relative expression, and green indicates low relative expression. (**B**): The scatter plot of the DEGs. Red dots represent up-regulated genes expressions, and green dots represent downregulated genes expressions. Data were log2(FC) transformed. (**C**): Venn diagram of the DEGs. (**D**): The Bar plots of enrichment analysis for the DEGs in different modules. The length of the bars indicates significance (−log10 transformed Benjamini–Hochberg adjusted FDR) and the enrichment moduli of the DEGs are shown on the right. (**E**): Network of DEGs associated with cell proliferation and cell cycle of mouse spermatogonia from three major GO. The target proteins are contained in the circle while the colour signifies the fold change of the DEGs. Furthermore, the number at the top right represents log2(FC) and the line is the strength of the protein interaction. (**F**): The expression analysis of *P21* between RNA-Seq and RT-qPCR in mouse spermatogonia. *Cdkn1a* also means the gene of *P21*.

To further analyse the role of TUBB4B in mouse spermatogonia, we used transcriptome sequencing to analyse gene expression in the control and experimental groups. A total of 675 differentially expressed genes (DEGs) were identified in *Tubb4b*-KO spermatogonia relative to controls, including 362 genes that were up-regulated and 313 genes that were down-regulated (Figure 6A–C). The significant DEGs between the control and experimental cells can be seen in the hierarchical clustering and scatter plot (Figure 6A,B). TUBB4B was showed to play an essential regulatory role in mouse spermatogonia.

To further investigate the role of TUBB4B in cell proliferation and cell cycle, we performed GO enrichment for 675 DEGs. The results showed that these DEGs were mainly concentrated in the processes affecting biosynthesis, metabolism, differentiation, cell conduction, the development of productive structure, and cell growth, among which three GO pathways, including negative regulation of cell population proliferation (GO:0008285), negative regulation of cell cycle G2/M phase transition (GO:1902750) and positive regulation of cell death (GO:0010942), were mainly associated with cell proliferation and cell cycle (Figure 6D). Furthermore, the *P21* gene (also known as *Cdkn1a*) was implicated in regulating these three pathways (Supplementary Table S2). After interacting the proteins in these three GO pathways, we found that the protein of CDKN1A, FOS, DDIT3, ESR1, CDC6, and FYN had strong interactions to affect cell cycle and proliferation (Figure 6E). Therefore, we detected that mRNA expression of *P21*, an important gene regulating cell proliferation and cell cycle after *Tubb4b* deletion, was increased in *Tubb4b*-KO cells and the result was consistent with transcriptome sequencing (Figure 6F). Abnormal changes in genes in these GO pathways may be the main reason for the slow growth of cells after TUBB4B deletion, and the cell cycle inhibitor factor *P21* plays a crucial role.

## 4. Discussion

Tubulin β-tubulin aggregation, migration and interaction with other related proteins occur during division of all mammalian cells and play a vital role in the stability of cell structure and cell proliferation cycle [16]. Although β-tubulin expression has been found to be abnormal in some pathological conditions [21], it is still difficult to accurately define its biology and function, especially at the stage of sperm development, because the reported research concentrates on the protein content. The observational results and β-tubulin contain a variety of isotype structures. In this study, we demonstrated that *Tubb4b* leads to disruption of mouse spermatogonial structure. In addition to the presumed preservation of the typical cell structure, their loss also leads to slow proliferation and cycle arrest of the spermatogonia.

Loss of *Tubb4b* leads to a decrease in cell proliferation, an increase in G1/0 population and a significant decrease in S population, implying that the loss of *Tubb4b* is not conducive to cell growth and cell cycle progression. Control cells showed faster growth than *Tubb4b*-KO cells. In addition, we have also proved that the *Tubb4b* gene is not essential for survival in mouse cells. Although it causes damage to cell structure, cells lacking *Tubb4b* are alive and there is no significant change in apoptosis, it only affects growth rate and cell cycle. Significant changes in gene expression can be found by sequencing, which not only alters the regulation of cellular amino acid synthesis and energy metabolism, but also reduces the regulation of cell population proliferation and cell cycle transition. It can be seen that TUBB4B is very important in the growth of mouse spermatogonia.

The complex of cyclin and cyclin-dependent kinases (CDKs) regulates the proliferation of mammalian cells. Cyclin D1 is a key regulator of cell cycle progression from G1 phase to S phase. Cell proliferation involves four cell cycle stages (G0/G1, S, G2 and M) [22]. The cell cycle process is regulated by two proteins namely cyclin and its kinase partner CDKs. Restriction transmission is coordinated by two cyclin families, namely the cyclin D family and the cyclin E family. The activation sequence is that D-type cyclin binds and activates CDK 4 and 6, then cyclin E activates CDK2 [23]. The S-phase kinase-related protein 2 (SKP2) cooperates with cyclins D and E to regulate cell cycle progression in G1-S phase [24]. Proper control of cell cycle progression depends on many factors. The cyclin-dependent kinase (CDK) inhibitor *P21* (also known as *Cdkn1a*) is one of the factors that promote cell cycle arrest in response to various stimuli. The inhibitory effect of P21 on cell cycle progression is related to its nuclear localization [25]. In spermatogonia lacking *Tubb4b*, *Cyclin D1* expression is significantly reduced, *P21* expression is significantly increased, and *Cdk4* expression is inhibited so that the binding efficiency of cyclin D1 and CDK is reduced. At the same time, SKP2 depletion leads to a decreased cell cycle proliferation rate and eventually prevents cells from the G1 phase from entering S phase. In the G1 phase.

Cytokines regulate the proliferation of mammalian cells. After resting cells were re-exposing with serum, expression of Cyclin *G-CSF* mRNA was significantly reduced in *Tubb4b* KO cells. Studies have reported that G-CSF can stimulate spermatogonia proliferation and protect spermatogenesis. The C/EBP is an essential regulatory protein in cell proliferation. Both C/EBP-a and -β can regulate cell proliferation and differentiation by inhibiting the action of the E2F complex [26]. In this study, the expression level of *C/EBP-a* was significantly reduced in spermatogonia lacking *Tubb4b*. Although *C/EBP-α* and *C/EBP-β* have a compensatory effect and the increase in *C/EBP-β* expression can compensate for deficiency of *C/EBP-α*, expression of *C/EBP-β* also decreased significantly in this study. This means that without *Tubb4b*, spermatogonia grow slowly and cell cycle disorders occur.

## 5. Conclusions

This study combined the CRISPR/Cas9 system to conditionally induce knockout of the mouse spermatogonia TUBB4B protein to construct a mouse spermatogonia cell line with *Tubb4b* gene knockout. We found that the loss of the *Tubb4b* gene significantly affected the proliferation and cell cycle of spermatogonia and, thus provided a cellular model and scientific basis for studying the function of *Tubb4b* gene in spermatogenesis.

## Data Availability

All data in this article are presented in the article, and the original data can be obtained by email asking the author.

## Supplementary Materials

The following supporting information can be downloaded at: https://www.mdpi.com/article/10.3390/genes13061082/s1, Table S1: The sequences of primers; Table S2: GO enrichment of DEGs in cell proliferation and cell cycle.
