# Supplementary material for: Tubulin TUBB4B Is Involved in Spermatogonia Proliferation and Cell Cycle Processes
Source: Genes (Basel). 2022 Jun 17;13(6):1082. doi: 10.3390/genes13061082 (PMC9222938; doi:10.3390/genes13061082)
Supplement: Supplementary file 1 [file genes-13-01082-s001.zip › genes-1742608-supplementary.pdf]

Supplementary Table S1

Supplementary Table S1. The sequences of primers.

Gene		Primer sequence（5’-3’）	Product size（bp）	Application
mU6	F：	TTTGGCGCCGGCTCGAGTGTACA	375	Vector
	R：	AAACAAGGCTTTTCTCCAAGGG		
*Tubb*4b-SgRNA1	F：	TGGAGAAAAGCCTTGTTTgCTACAACGAAGCCACCGGTAGTTTTAGAGCTAGAAATAGC		Vector
*Tubb*4b-SgRNA2	F：	TGGAGAAAAGCCTTGTTTgATGGGGTAGAGGAGCCTTACGTTTTAGAGCTAGAAATAGC	163	Vector
*Tubb*4b-SgRNA3	F：	TGGAGAAAAGCCTTGTTTgGGGTAGAGGAGCCTTACCGGGTTTTAGAGCTAGAAATAGC		Vector
SgRNA	R：	CACCGGTTAGCGCTAGCTAATGCC		Vector
*Cas*9	F：	GGTATCCACGGAGTCCCAGCAGCC	4176	Genome
	R：	TTACTTTTTCTTTTTTGCCTGGCCGG		
*Tubb*4b	F：	GGGAGGTAATCAGCGACGAG	464	Genome
	R：	CCGCAGTCAGTTGGACCTTC		
*P*21	F：	CCAAGATAGCCGAGTTCA	115	qRT-PCR
	R：	ACAACCAGGAAGACGACA		

Note: F means forward primer, and R means reverse primer. The gene sequence of *Gapdh*, *C/ebp α*, *C/ebp β*, *G-csf*, *Cdk 2*, *Cdk 4*, *Cyclin D1*, *Cyclin E* and *Skp2*, were the same as previously described (Feng, M.; Bai, Y.; Chen, Y.; Wang, K. Knockout of the Transducin-Like Enhancer of Split 6 Gene Affects the Proliferation and Cell Cycle Process of Mouse Spermatogonia. *Int. J. Mol. Sci.* **2020**, *21*, doi: 10.3390/ijms21165827.).

Supplementary Table S2

Supplementary Table S2. GO enrichment of DEGs in cell proliferation and cell cycle.

Name	ID	enriched genes
negative regulation of cell population proliferation	GO:0008285	Cdkn1a, Ackr3, Ctsl, Esr1, Fas, Fosl1, Gdf11, Klf4, Lif, Meis1, Ppp1r15a, Nos3, Nppb, Etv4, Pmp22, Ptprm, Rbp4, Rps6ka2, Ccl2, Sfrp4, Slc16a2, Slfn2, Snai2, Sox11, Tgfbr3, Tgif1, Tnfaip3, Tnfrsf9, Vdr, Ceacam1, Ndrg2, Nupr1, Slurp1, Cd274, Trp53inp1, Trim35, Dhcr24, Il33, Atf5, Cth, Trib1, Kctd11, Fbxo2, Sulf1, Chd5
negative regulation of cell cycle G2/M phase transition	GO:1902750	Ccng1, Cdkn1a, Fhl1, Hmga2, Ier3, Orc1, Cdc6, Atf5, Clspn, Brsk1
positive regulation of cell death	GO:0010942	Atf3, Bnip3, Cdkn1a, Clu, Gadd45a, Ddit3, Fas, Fos, Fosl1, Fyn, Hmga2, Hp, Mmp3, COX2, Ppp1r15a, Nqo1, Mycn, Nos2, Nos3, Pik3cd, Rps6ka2, Ccl2, Sfrp4, Plscr1, Unc13b, Unc5c, Vdr, Ceacam1, Irf5, Htra1, Nupr1, Cd274, Trp53inp1, Trim35, Ecscr, Fam162a, Akap12, Acox2, Bmf, Zc3h12a, Nfkbid
